# Relationship Between Health-Related Behaviors and Family Quality of Life in Children with Autism Spectrum Disorder

**DOI:** 10.1007/s10803-025-06900-1

**Published:** 2025-06-04

**Authors:** Dunia Garrido, M. Mar Gomez-Perez, Dafina Petrova, Miriam Arreola, Andres Catena, Rocio Garcia-Retamero

**Affiliations:** 1 Department of Developmental and Educational Psychology, University of Granada, Granada, Spain; 2 Departamento de Psicología Evolutiva y de la Educación, Universidad de Málaga, Granada, Spain; 3 Instituto de Investigacion Biosanitaria ibs. GRANADA, Granada, Spain; 4 Escuela Andaluza de Salud Publica (EASP), Granada, Spain; 5 CIBER of Epidemiology and Public Health (CIBERESP), Madrid, Spain; 6 University of Granada, Granada, Spain; 7 Department of Experimental Psychology, University of Granada, Granada, Spain; 8 Max Planck Institute for Human Development, Berlin, Germany

**Keywords:** Autism spectrum disorder, Family quality of life, Sleep, Mealtime, Screen time, Physical activity

## Abstract

Improving family quality of life (FQoL) of families of children with autism spectrum disorder (ASD) is a key priority in clinical research and practice. Previous studies have suggested that certain health-related behaviors in children with ASD, such as sleep or physical activity, may affect FQoL. However, the relative and independent effects of different health-related behaviors on FQoL remain poorly understood. This study investigated the relationship between sleep, mealtime difficulties, screen time, physical activity, and FQoL in ASD. Parents of 65 children with ASD aged four to thirteen years completed a detailed assessment of their children’s health-related behaviors and FQoL. A multiple regression framework was used to estimate the independent contribution of each health-related behavior to FQoL. Only sleep and mealtime problems were independently associated with lower FQoL, even after controlling for physical activity, screen time, gender, age, traits of ASD, and intelligence, accounting for a 60, and 3% of the variance, respectively. All categories of sleep and mealtime problems were associated with FQoL, with particularly strong correlations for sleep anxiety (*r* =.71), daytime sleepiness (*r* =.70), and food refusal (*r* =.72). In addition to core autistic features, sleep and mealtime behaviors are associated with FQoL in children with ASD. Interventions aimed at improving sleep habits and reducing mealtime difficulties many enhance FQoL in children with ASD.

Autism spectrum disorder (ASD) is a neurodevelopmental condition with a prevalence in children ranging from 1 in 54 (Maenner et al., 2020) to 1 in 100 (Zeidan et al., 2022). This condition is characterized by persistent deficits in social interaction skills and social communication (both verbal and nonverbal), alongside repetitive, restrictive, and stereotyped behavior, interests, or activities (American Psychiatric Association [APA], 2022). In addition to these core features, ASD can also affect health related behaviors such as sleep, mealtime behaviors, screen time, and physical activity with implications for broader life functioning, including individual and family quality of life (FQoL) (Delahaye et al., 2014; Jones et al., 2017; Kuhlthau et al., 2018; Mannion & Leader, 2014; Margari et al., 2020; Slobodin et al., 2019). For instance, more behavioral problems have been consistently associated with lower quality of life (including health related quality of life) in children with ASD (Bolbocean et al., 2023; Coales et al., 2019; Delahaye et al., 2014; Ikeda et al., 2014; Kose et al., 2013; Kuhlthau et al., 2018; Meral & Fidan, 2015; Potvin et al., 2015).

Most previous research has focused on the quality of life of children or parents, usually expressed as the impact of a health condition on an individual’s QoL (i.e., health-related quality of life), satisfaction with several aspects of life or level of engagements in different types of activities (Kuhlthau et al., 2018). In contrast, much less is known about FQoL. FQoL is an indicator of the satisfaction of families who have a child with a disability (Zeng et al., 2021). FQoL typically includes five components (i.e., family interaction, parenting, emotional well-being, physical/material well-being, and disability support), which together describe the extent to which family needs are met, the enjoyment of time together, and the opportunity to engage in meaningful activities (Park et al., 2003). In recent years, there has been a growing interest in identifying health-related behaviors that may influence or protect FQoL in ASD (Binnendyk & Lucyshyn, 2009; Cardy et al., 2021; Connor et al., 2023; Liu et al., 2021; Logrieco et al., 2022; Losada-Puente et al., 2022; Sahan et al., 2022). Most studies to date have investigated the influence of single health-related behaviors, without providing a broader picture by considering a broader behavioral profile including diverse behaviors with potential relevance to FQoL. To address this gap, the present study examines the individual contribution of diverse health-related behaviors on FQoL, including sleep habits, mealtime behavior, screen time, and physical activity.

## Sleep Habits and FQoL

Children with ASD experience a higher incidence of sleep problems compared to typically developing peers, ranging from 40 to 80% (Cohen et al., 2014). These sleep problems include bedtime resistance, problems falling asleep, problems staying asleep, frequent and prolonged night-time awakenings, reduced sleep duration, parasomnias, and daytime sleepiness (Diaz-Roman et al., 2018; Liu et al., 2021). Such challenges are often exacerbated by core ASD symptomatology and co-occurring behavioral problems (Mazurek & Sohl, 2016; Veatch et al., 2017). As a result, these sleep problems negatively impact both child and family functioning, thereby impacting FQoL.

For example, some studies have shown a negative relationship between sleep problems and multiple aspects of FQoL in children with ASD and their families (Delahaye et al., 2014; Kuhlthau et al., 2018; Liu et al., 2021); this pattern differs from that observed in typically developing peers (Liu et al., 2021). Furthermore, when comparing children with ASD who do and do not have sleep problems, parents of the former report lower physical and psychological well-being (Mannion & Leader, 2023). Various intervention programs have been introduced to address these sleep problems. Both melatonin (Schroder et al., 2019) and behavioral interventions (Papadopoulos et al., 2022) have shown how improvements in children´s sleep have led to better well-being for both child and caregivers, as well as enhanced FQoL (Papadopoulos et al., 2022; Schroder et al., 2019).

## Mealtime Behavior and FQoL

In addition to sleep habits, difficulties during mealtimes that children with ASD often exhibit (Margari et al., 2020) is another domain which can significantly impact FQoL (Connor et al., 2023; Leader et al., 2022; Meral & Fidan, 2015). These difficulties an stem from core features of ASD, such as repetitiveness, ritualistic behaviors, inflexibility, and hypo- or hyper-responsiveness to sensory stimuli (Margari et al., 2020). They have been reported in a large proportion of children with ASD, ranging from 31 to 84% (Mayes & Zickgraf, 2019), and typically include selective intake (food selectivity, picky eating, food neophobia/aversion), restricted appetite, and fear of feeding (Kerzner et al., 2015; Margari et al., 2020).

In these sense, studies reveal that feeding and mealtime challenges are associated with lower QoL in both children and families. Conversely, improvements in these behaviors are associated with higher FQoL (Connor et al., 2023; Leader et al., 2022; Meral & Fidan, 2015). A notable example is the study conducted by Binnendyk and Lucyshyn (2009), which shows that addressing food refusal in children with ASD can lead to better eating behaviors and improved FQoL.

## Screen Time and FQoL

Another relevant factor are high levels of screen time in which children with ASD are often engage (Bolbocean et al., 2023; Cardy et al., 2021; Folostina et al., 2023). Screen-based activities (i.e., watching TV, using a mobile phone, or playing computer/video games) can match their preference for lower social demands, making these activities comparatively appealing (Cardy et al., 2021; Montes, 2016; Slobodin et al., 2019; Stiller & MöBle, 2018). Parents may also use screens to manage children’s behavior by providing a less intimidating interactive environment (Matheson & Douglas, 2017). Although this can be beneficial, excessive screen time has been linked to negative health effects, such as obesity or sleep problems (Dong et al., 2023; Healy et al., 2017).

Regarding the impact of screen time on FQoL in ASD, studies show inconsistent findings. For instance, Folostina et al. (2023) show a positive correlation, suggesting that moderate screen use can help calm children and may thus be beneficial for families. In contrast, Bolbocean et al. (2023) found that more TV viewing correlated with lower FQoL and increased behavioral problems. Similarly, Cardy et al. (2021) reported a negative impact of screen time (i.e., playing video games or watching videos) on both FQoL and mental health in boys with ASD, although the effect was positive when screen time involved educational games, using therapeutic apps, or online social interaction with friends and family.

## Physical Activity and FQoL

With regard to physical activity as another important health-related behavior related with FQoL, children with ASD often have fewer opportunities for physical activity, lower levels of participation and poorer performance compared to their peers (Pan et al., 2016; Pavlova et al., 2021; Sahan et al., 2022). Possible reasons include social deficits (APA, 2022), high prevalence of motor impairments (up to 83%; Hilton et al., 2011), and lower overall motor coordination (Fournier et al., 2010; Pavlova et al., 2021). However, despite the difficulties, physical activity can benefit this population by improving motor skills, reducing behavioral problems, and facilitation social opportunities and FQoL (Huang et al., 2020; Sorensen & Zarrett, 2014).

Concerning relation with FQoL, research shows that children with ASD who engage in higher levels of physical activity enjoy better FQoL (Logrieco et al., 2022; Sahan et al., 2022). It is therefore not surprising that children with ASD often report lower FQoL than peers, given their lower physical fitness (Pavlova et al., 2021). Consequently, multiple studies have targeted physical activity as an intervention. For example, Jimeno (2019) showed that a sport-based program improved motor skills, wellbeing and FQoL in children with ASD. Similarly, Toscano et al. (2018) informed that a 48-week exercise-based intervention led to improvements in metabolic indicators, autism traits, and FQoL. Furthermore, Zhao et al. (2021) observed that web-based parent–child physical activity improved parents´ mental health and FQoL. Taken together, these improvements in FQoL resulting from physical activity underscore their strong association.

### The Current Study

Taken together, these findings highlight the multifaceted ways in which health-related behaviors (i.e., sleep habits, mealtime behavior, screen time, and physical activity) may contribute to FQoL in children with ASD. Nevertheless, it is unknown to what extent there behaviors may have unique relationships with FQoL, because to the best of our knowledge, no research has studied them jointly and examined their independent effects. Existing research often considers a subset of these variables, alongside autism traits, cognitive ability, internalizing/externalizing behaviors, mental health, or sociodemographic factors (Eapen et al., 2024; Kuhlthau et al., 2018). Only a few works (e.g., Kong et al., 2023; Li et al., 2022), have examined combinations of physical activity, sedentary behavior, and sleep, suggesting that adherence to integrated 24-hour movement guidelines (Tremblay et al., 2016, cited by Li et al., 2022) is beneficial for FQoL. Furthermore, while some studies have looked at different health-related behaviors, they have not determined the extent to which each behavior independently contributes to FQoL, which prevents the identification of priority areas for intervention. Given that health behaviors are potentially modifiable, understanding which behaviors have the greatest impact on FQoL would allow more effective and targeted intervention strategies to be developed. However, there is insufficient evidence in the literature to suggest which behaviors should be prioritized in family support programs.

The present study aims to provide evidence that could be informative for the design of interventions aiming to improve the quality of life of families of children with ASD. In addition, the results of this study could guide clinical decision making. For example, it might be meaningful to focus first on health-related behaviors with greater impact on FQoL.

Therefore, the present study seeks to expand the literature by investigating how FQoL relates to these health-related behaviorssleep habits, mealtime behavior, screen time, and physical activity. In addition, given the substantial impact of isolated health-related behaviors on FQoL, we hypothesize that these health-related behaviors would explain significant variance in FQoL, even after controlling for age and autistic traits (i.e., level of support needed).

## Methods

### Participants

We recruited a total of 70 families with a child with ASD. Of these, 65 families completed the evaluation and were included in this study. These families had a child with ASD (mean age of 6.76, SD = 2.22; range = 4.4–13.00) and were recruited through several ASD-focused parent associations that we approached to invite them to take part in this study. Most study participants were male (*n* = 57; 86.4% male). Information regarding additional sociodemographic variables is included in Table 1. The inclusion criteria were: having received the ASD diagnosis based on DSM-IV-TR (APA, 2000) and/or DSM-5 (American Psychological Association [APA], 2022) criteria, having a level of nonverbal intelligence within the range considered normative, not having any formally diagnosed comorbid conditions, and being enrolled in school. In addition, the diagnosis was verified by the Gilliam Autism Rating Scale, third edition (GARS-3; Gilliam, 2014).

**Table 1:** Sociodemographic and autism-related characteristics of study sample (*n* = 65)

Variable	Mean (SD) or *N*, %	Missing (*N*, %)
Child gender - male	57, 87.69%	0, 0%
Child age	7.99 (± 2.44)	0, 0%
Child age at diagnosis	2.80 (± 1.44)	0, 0%
Child IQ	95.21 (± 11.15)	17, 26.15%
Child speech development - non-verbal	5, 7.69%	0, 0%
Autism traits score	96.29 (± 16.62)	0, 0%
Autism level according to DSM-5		0, 0%
Level of support 1	5, 7.69%	
Level of support 2	37, 56.92%	
Level of support 3	23, 35.39%	
Siblings		0, 0%
None	16, 24.6%	
One sibling	34, 52.3%	
Two or more siblings	15, 23.1%	
Parent relationship status		0, 0%
Unpaired (i.e., single)	6, 9.09%	
Unpaired (i.e., divorced)	3, 4.55%	
Married	56, 86.15%	
Primary caregiver – mother	56, 86.15%	0, 0%
Primary caregiver education		0, 0%
Secondary school	26, 40.00%	
High/vocational school	14, 21.54%	
Some college or more	25, 38.46%	
Primary caregiver employment status		0, 0%
Unempoyed	23, 35.38%	
Part time job	24, 36.36%	
Full time job	18, 27.27%	

### Measures

All families completed a demographic questionnaire developed for the current study, which included family structure, child and parents’ ages, gender, levels of education, and employment status. Additional specific measures related to autistic traits, nonverbal intelligence, health-related behaviors, and FQoL were also collected and described below. Importantly, all measures of health-related behavior are based on activities and patterns over 7 days in a week that had no holidays or other elements that made it particularly different from what might be considered a typical week for these families.

### Autistic Traits

*Gilliam Autism Rating Scale*,* Third Edition* (GARS-3; Gilliam, 2014). This scale evaluates six ASD-related domains: (1) Restricted/repetitive behaviors, (2) social interaction, (3) social communication, (4) emotional responses, (5) cognitive style, and (6) maladaptive speech. This instrument provides an overall measure of autistic traits and was used here to confirm the ASD diagnosis. Moreover, GARS-3 generates an ASD index that indicates the likelihood of ASD and support levels based on DSM-5 (APA, 2022). This instrument demonstrates good internal consistency, with a global test-retest reliability of 0.90, and Cronbach’s alpha scores ranging from 0.79 to 0.95 (Gilliam, 2014). In our sample, all subscales showed good internal consistency: specifically, Cronbach’s alpha = 0.88 for restricted/repetitive behaviors, 0.88 for social interaction, 0.85 for social communication, 0.85 for emotional responses, 0.69 for cognitive style, and 0.77 for maladaptive speech.

### Nonverbal Intelligence

*Raven’s Progressive Matrices - Second edition* (Raven’s 2; Raven et al., 2018). This individually administered, non-verbal assessment of general cognitive ability is designed for individuals aged 4 to 90 years. It assesses cognitive functions such as perception, attention to visual detail, inductive reasoning, fluid intelligence, broad visual intelligence, classification, spatial ability, simultaneous processing, and working memory. The digital short form was selected for the current study. Raven’s 2 shows good internal consistency, with a global test-retest reliability of 0.85 and Cronbach’s alpha values of 0.80 for children aged 4 to 10 years (McLeod & McCrimmon, 2021).

### Health-Related Behaviors

*Children’s Sleep Habits Questionnaire* (CSHQ; Owens et al., 2000). This 33-item parent-report scale assesses sleep problem severity in school-aged children. It comprises eight subscales: `bedtime resistance´, `sleep onset delay´, `sleep duration´, `sleep anxiety´, `night wakings´, `parasomnias´, `sleep disordered breathing´, and `daytime sleepiness´. The total score is the sum of all responses, and higher scores indicate the worse sleep habits. A cutoff of 41 was used to determine clinically significant sleep problems (Leader et al., 2022; Owens et al., 2000). Although normed on neurotypical children aged from 4 to 10 years, this questionnaire has been evaluated with multiple cohorts, including children with ASD (Liu et al., 2021), as well as Spanish samples (Lucas-de la Cruz et al., 2016). It demonstrates good reliability, with a Cronbach’s alpha of 0.81 for the full Spanish version, showing similar psychometric properties to other country versions (Lucas-de La Cruz et al., 2016). In our sample, all subscales showed good internal consistency, which Cronbach’s alpha = 0.90 for bedtime resistance, 0.89 for sleep duration, 0.81 for sleep anxiety, 0.74 for night wakings, 0.51 for parasomnias, 0.82 for sleep disorder breathing, and 0.80 for daytime sleepiness.

*Brief Autism Mealtime Behavior Inventory* (BAMBI; Lukens & Linscheid, 2008). This 18-item questionnaire assesses food selectivity and mealtime behavior across three factors: “limited variety”, “food refusal”, and “autism characteristics”. The total score reflects eating difficulties, with higher total scores indicating more problematic mealtime behavior. A cutoff of 34 was used to determine the presence of feeding and mealtime problems. This test shows good internal consistency, with Cronbach’s alpha scores of 0.88 (Lukens & Linscheid, 2008), and it is widely used to assess feeding difficulties in ASD (see Page et al., 2021 for a systematic review). In our sample, all factors demonstrated good internal consistency, as indicated by Cronbach’s alpha values of 0.89 for limited variety, 0.88 for food refusal, and 0.52 for autism characteristics respectively.

*Screen time*. To estimate each child’s daily screen time, parents completed a questionnaire developed by our research team, based on scales widely used in the literature in children with ASD (Dong et al., 2021; Healy et al., 2017; Heffler et al., 2022; see Slobodin et al., 2019 for review). We assessed screen time on both weekdays and weekends using for questions: “On a normal day, about how many hours does your child spend watching television?”, “On a normal day, about how much time does your child spend using the computer or tablet?”, “On a normal day, about how much time does your child spend using the smartphone?”, and “On a normal day, about how much time does your child spend playing video games?”. Following Dong et al. (2021), the average daily screen time (in minutes) was calculated with the formula:

$$\eqalign{& ({\rm{screen}}\>{\rm{time}}\>{\rm{per}}\>{\rm{day}}\>{\rm{on}}\>{\rm{weekdays}} \times {\rm{5}}) \cr & {\rm{ + }}({\rm{screen}}\>{\rm{time}}\>{\rm{per}}\>{\rm{day}}\>{\rm{on}}\>{\rm{weekends}} \times {\rm{2}}) \div 7 \cr} $$

where higher scores reflect greater screen use. Additionally, following Heffler et al. (2022), we examined direct viewing vs. background exposure, type of content (child-oriented vs. adult-oriented), and whether the child was typically accompanied during screen use.

*Physical Activity Questionnaire for Children* (PAQ-C; Kowalski et al., 2004). This 9-item, 7-day recall instrument is intended for elementary and middle school children and covers different activities and sports, as well as physical education class effort, lunch breaks, after-school time, and weekend routines. The final PAQ-C activity summary score (ranging from 0 to 5) is obtained by averaging the 9 items, with higher scores indicating better physical activity habits. The PAQ-C has been applied in diverse samples, including children with ASD (Bricout et al., 2018) and Spanish populations (Benitez-Porres et al., 2016). This scale shows a moderate-to-high reliability when compared to accelometro-based measures (Voss et al., 2017), and is considered acceptable in terms of reliability and validity for children with ASD. Cronbach’s alpha ranging from 0.76 to 0.83 have been reported for the Spanish PAQ-C version, consistent with other versions (Benitez-Porres et al., 2016). In our sample, this scale showed good internal consistency (i.e., Cronbach’s alpha = 0.87).

### Family Quality of Life

*Family Quality of Life Survey* (Spanish version, FQoL; Verdugo et al., 2009). This questionnaire yields two global scores (i.e., importance of FQoL and satisfaction with FQoL) based on five factors: emotional wellbeing, family interaction, financial resources, the role of father/mother, and physical wellbeing. It is adapted from the Family Quality of Life Survey, developed at the Beach Center on Disability, Kansas (USA) (Hoffman et al., 2006), and has demonstrated good internal consistency, with Cronbach’s alpha scores of 0.88 and 0.85 for importance and satisfaction, respectively. In our sample, the satisfaction scale showed good internal consistency (Cronbach’s alpha = 0.82).

### Procedure

The final version of the project and questionnaires is available as supplementary material on the Open Science Framework (https://osf.io/p4c6v/). A priori power analysis (using G*Power calculator; Faul et al., 2007) showed that with a total of eight predictors, an assumed power = 0.95, alpha = 0.05, and effect size d = 0.20, the minimum sample size needed was 56 participants.

The study was approved and conducted in accordance with the ethical standards of the Provincial Ethics in Biomedical Research Committee of Granada (Reference: 0355-N-23). All parents signed informed consent forms prior to their child’s participation. Evaluations were carried out in one or two sessions, depending on each family’s need.

Families were recruited through local ASD-focused parent associations that provide support and resources for children with autism and their families. The researchers contacted these associations via email, briefly describing the purpose of the study and its inclusion criteria (e.g., child age range and ASD diagnosis). Association representatives then shared the invitation with their members through newsletters and scheduled group meetings. Participation was voluntary and no incentives were offered; confidentiality and anonymity were ensured in accordance with institutional ethical guidelines. This strategy facilitated access to families already engaged in ASD-related support networks, although it may introduce a selection bias toward families who are more actively involved in community services.

### Data Analysis

Analyses were conducted in JASP, version 0.19 for Mac (JASP Team, 2024). First, to determine the relationship between the FQoL and potential health-related behaviors in children with ASD (i.e., sleep habits, mealtime behavior, screen time, and physical activity), we ran bivariate Pearson correlations.

Next, to assess the independent effects of children’s health-related behaviors on FQoL, we performed a multiple linear regression. Specifically, the satisfaction score from the FQoL questionnaire was the dependent variable, while age, autistic traits (GARS-3), and any health-related behaviors significantly correlated with FQoL satisfaction where included as independent variables. Participants needed complete data on all variables in the regression model, except intelligence, for which missing data were imputed with the group mean (*N* = 8). For variables that explained variance in FQoL, *f*^2^ was used as a measure of effect size; considering scores of 0.02, 0.15, and 0.35 as small, medium, and large effect sizes, respectively (Cohen et al., 2003). Additional analyses were also conducted to examine which specific sub-components of the significant health-related behaviors were most strongly linked to FQoL. Only variables significantly correlated with FQoL were included as predictors. We computed variance inflation factor (VIF) to ensure no issues with multicollinearity, using the range of 1–10 as indicative of its absence (Cohen et al., 2003).

## Results

In line with our main goal of examining whether sleep problems, mealtime difficulties, screen time, and physical activity independently predict FQoL, we tested the hypothesis that these health-related behaviors would explain a significant amount of variance in FQoL, even after controlling for age and autistic traits. We begin this section by describing the prevalence and descriptive statistics of these key variables, followed by the correlational analyses that assess their bivariate relationships with FQoL. Finally, we report the results of the regression models to evaluate the unique contribution of each predictor to FQoL.

Over 60% of children with ASD (*n* = 44, 67.69%) had sleep problems on the CSHQ (see Table 2). In this sample, daytime sleepiness was the most frequently reported sleep problem, followed by bedtime resistance and parasomnias. The mean BAMBI total score was 44.45, indicating that the average score exceeding the threshold for feeding and mealtime problems. More specifically, only 21 participants (32.31%) scored below the clinical cutoff. The majority of children with ASD (*n* = 53, 81.54%) spent fewer than two hours per day in front of a screen. Regarding physical activity, 24 children (36.92%) performed activities not listed in the PAQ-C. Most of these 24 children jumped on trampolines (*n* = 20), whereas 2 engaged in rock climbing, and another 2 did multi-sport activities (i.e., adapted sports). Finally, FQoL results from caregivers showed an average global satisfaction score was 4.08, reflecting generally high satisfaction.

**Table 2:** Descriptive statistics of health-related behaviors and FQoL

Variable	Mean	SD	Min-max	Range
Health-related behaviors				
Sleep problems	51.677	16.809	33.00–86.00	33.00–99.00
Bedtime resistance	10.00	4.142	6.00–18.00	6.00–18.00
Sleep onset delay	1.831	0.945	1.00–3.00	1.00–3.00
Sleep duration	4.815	2.704	3.00–9.00	3.00–9.00
Sleep anxiety	6.923	2.890	4.00–12.00	4.00–12.00
Night wakings	4.538	1.777	3.00–9.00	3.00–9.00
Parasomnias	9.785	2.342	7.00–15.00	7.00–21.00
Sleep disordered breathing	4.477	1.872	3.00–8.00	3.00–9.00
Daytime sleepiness	12.585	3.733	8.00–20.00	8.00–24.00
Mealtime problems	44.446	17.089	18.00–84.00	18.00–90.00
Limited variety	24.569	9.970	8.00–39.00	8.00–40.00
Food refusal	10.000	5.728	5.00–24.00	5.00–25.00
Autism characteristics	9.877	3.710	5.00–21.00	5.00–25.00
Screen time	73.702	60.819	7.50–220.71	
Physical activity	2.550	1.310	0.00–4.52	0.00–5.00
Family quality of life				
Importance - global score	4.643	0.312	4.02–5.00	1.00–5.00
Satisfaction - global score	4.038	0.638	2.50–5.00	1.00–5.00

Note: Screen time = total amount of min/day

Bivariate Pearson correlations with satisfaction on the FQoL scale are shown in Table 3. Higher satisfaction was associated with fewer sleep and mealtime problems, as well as daily screen time. However, it was not associated with physical activity. In addition, families with older children showed higher satisfaction with FQoL.

**Table 3:** Pearson’s correlation statistics for potential predictors of family quality of life (FQoL)

	1	2	3	4	5	6	7	8
1. Family Quality of Life								
2. Sleep problems	− 0.775*							
3. Mealtime problems	− 0.747*	0.851*						
4. Screen time	0.374*	− 0.470*	− 0.333*					
5. Physical activity	0.041	0.014	− 0.097	− 0.069				
6. Child’s age	0.270*	− 0.301*	− 0.233	0.166	− 0.094			
7. Autistic traits	− 0.193	0.221	0.155	− 0.233	0.105	− 0.157		
8. Non-verbal intelligence	0.072	− 0.064	− 0.196	− 0.038	0.056	− 0.066	− 0.172	

Note: *p <.05

For our main analysis, we conducted a linear regression with FQoL satisfaction as the target variable. The remaining variables (i.e., sleep problems, mealtime problems, screen time and child’s age) were entered as predictors, while gender, intelligence, and autistic traits (level of support) were included as control variables. There was no multicollinearity among these predictors, as indicated by the variance inflation factors (VIF), ranging form 1.057 to 2.095, and tolerance values between 0.477 and 0.933.

The results of the regression are presented in Table 4, including standardized regression coefficients (βs) and each predictor change in R^2^. Overall, the model explained 63% of the variance in FQoL, *F* (4, 60) = 25.688, *p* <.001. Examination of the beta coefficients revealed that sleep problems and mealtime problems were both significant contributors to the model, accounting for 60% and 3% of the variability, respectively. Screen time, age, gender, autistic traits, and nonverbal intelligence did not significantly contribute to the model (*ps* > 0.05).

**Table 4:** Multiple regression analysis for variables predicting Family Quality of Life (FQoL)

						95 CIs	
Predictor	Β	SE	*Β*	*P*	R^2^	*Lower*	*Upper*
Sleep problems	− 0.017	0.006	− 0.460*	0.007	0.600	− 0.030	− 0.005
Mealtime problems	− 0.012	0.006	− 0.331*	0.032	0.028	− 0.024	− 0.001
Screen time	0.001	0.001	0.039	0.662	0.001	− 0.001	0.002
Child’s age	0.012	0.022	0.047	0.569	0.002	− 0.031	0.055

Note: *p <.05

Follow-up analysis (see Table 5) showed that FQoL was significantly correlated with all subscales of the sleep problems measure (i.e. bedtime resistance, sleep onset delay, sleep duration, sleep anxiety, night wakings, parasomnias, sleep-disordered breathing, and daytime sleepiness), with correlation values ranging from − 0.58 to − 0.71. Likewise, there were significant correlations between FQoL and all factors measuring feeding difficulties (i.e. limited variety, food refusal, and autistic characteristics), ranging from − 0.63 to − 0.72. Thus, all types of difficulties appeared to be similarly related to FQoL.

**Table 5:** Pearson’s correlation between sleep and mealtime problems and family quality of life (FQoL)

	1. Family Quality of Life
1. Family Quality of Life	
Sleep problems	
2. Bedtime resistance	− 0.683*
3. Sleep onset delay	− 0.616*
4. Sleep duration	− 0.666*
5. Sleep anxiety	− 0.712*
6. Night wakings	− 0.578*
7. Parasomnias	− 0.607*
8. Sleep disordered	− 0.662*
9. Daytime sleepiness	− 0.703*
Mealtime problems	
10. Limited variety	0.628*
11. Food refusal	0.717*
12. Autism characteristics	0.658*

Note: *p <.05

## Discussion

To the best of our knowledge, this is the first study to examine the unique associations of multiple health-related behaviors (i.e., sleep habits, mealtime behavior, screen time, and physical activity) with FQoL in a Spanish sample of children with ASD. Our study identifies sleep and mealtime challenges as primary modifiable factors with potential impact on FQoL, independent of demographic and clinical variables (such as autistic traits). Whereas screen time had a positive relationship with FQoL, no relationship was detected for physical activity. Coupled with previous evidence on behavioral correlates of family well-being (Cohen et al., 2014; Connor et al., 2022), these findings highlight the needed for interventions targeting sleep hygiene and/or feeding behaviors.

With regard to the health-related behaviors observed in our sample, the results showed findings consistent with prior studies. Specifically, 67% of children with ASD experienced sleep problems, which aligns with the incidence reported in previous investigations (Cohen et al., 2014) and is 20% higher than that reported in others (e.g., Liu et al., 2021). Although the underlying causes of these prevalent sleep problems remain unclear, research suggests potential neurobiological causes (e.g., maturational differences and abnormal melatonin production) (Souders et al., 2017). In addition to neurobiological factors, such as maturational differences and abnormal melatonin production, environmental factors, such as inconsistent bedtime routines or exposure to screens during the night-time hours, have been demonstrated to exacerbate sleep problems in children with ASD (Richdale & Schreck, 2019). Such findings are also partially reflected in the current study, in terms of results regarding bedtime resistance. In terms of mealtime behavior, 32% of children with ASD exhibited feeding or eating problems, a figure consistent with prior work in ASD populations (Mayes & Zickgraf, 2019). Meanwhile, 82.5% of children spent less than two hours per day in front of a screen, aligning with a recent systematic review in typically developing children, where average daily screen time was 2.77 h (Qi et al., 2023). Finally, the physical activity scores were comparable to normative values contrary to other studies (Pan et al., 2016; Pavlova et al., 2021; Sahan et al., 2022) possibly due to structured daily routines of our participants, including school hours.

Nevertheless, studies examining multiple health-related behavior and their combined effects on FQoL in ASD or other neurodevelopmental populations are scarce. The present study addressed how sleep and mealtime problems, screen time, and physical activity collectively influence FQoL. We found that both sleep and mealtime problems were risk factors for lower FQoL, independent of other variables such as gender, age, and autistic traits. These findings support and further elucidate previous research. For example, Delahaye et al. (2014), Kuhlthau et al. (2018), and Liu et al. (2021) demonstrated a negative correlation between sleep problems and parental quality of life, while other studies (e.g. Connor et al., 2023; Leader et al., 2022; Meral & Fidan, 2015) have reported that reductions in mealtime difficulties can predict increases in FQoL.

Despite these established links, our follow-up analyses did not reveal any specific subcomponents if sleep or mealtime problems as uniquely predictive of FQoL. This may be due to fact that the tests used are specifically designed for the ASD population, with items reflecting various aspects observed in this population. However, anxiety and daytime sleepiness appeared somewhat more salient than night wakings. Meanwhile, in the domain of mealtime problems, that food refusal may have a stronger influence on FQoL than other factors.

In contrast, although screen time did not emerge as a significant predictor in the main regression analyses, it showed a positive correlation with FQoL. In this sense, the findings of this study are consistent with Folostina et al. (2023), who reported a positive association between screen time and FQoL, suggesting that moderate screen use might benefit families of children with ASD. A plausible explanation is that screen use can sometimes help manage or regulate challenging behavior (Matheson & Douglas, 2017).

Interestingly, our results indicated that physical activity in children with ASD did not affect FQoL satisfaction. This contrasts with prior studies (Logrieco et al., 2022; Sahan et al., 2022), which found increases in FQoL linked to higher physical activity. The PAQ-C questionnaire reports general level of moderate to vigorous physical activity, not the amount of time spent doing physical activity. This limits the comparability of our results to previous studies that reported poor adherence to daily physical activity recommendations in children with ASD (i.e., 60 min per day; Case et al., 2020). The results for our sample show lower levels than those reported in other studies with samples of Spanish children with typical development samples (Benitez-Porres et al., 2016), but similar to other studies including children with ASD (Bricout et al., 2018). A plausible explanation in line with our earlier discussion is that school-aged children with ASD often follow a structured program, so that there is limited variability in activity levels between weekdays. It would therefore be plausible to asume that they engage in similar levels of physical activity to other school-aged children with ASD. For instance, the structured routines in special education schools often including scheduled physical activity or adapted physical education may standardize participation levels among children with ASD.

### Limitations and Future Studies

Despite these contributions, our study has several limitations that should be considered when interpreting these results. First, although parent-reported measures are valid in pediatric populations (Delahaye et al., 2014), future research should explore self-reported measures when possible. Although some studies have suggest substantial agreement between parental report and objective measures (e.g., accelerometers for physical activity; Voss et al., 2017), the development of suitable objective measures for children with communication difficulties (common in ASD) remains an important area for exploration.

Second, although the gender ratio in our sample reflects commonly reported male predominance in ASD, future studies should include more female participants to enhance generalizability. In addition, future research can also broaden the sample composition by considering more diverse socioeconomic and cultural contexts. Exploring a variety of income levels, family dynamics, and geographical backgrounds would allow for more representative findings and enhance the ecological validity of the conclusions. Moreover, this approach would provide a more inclusive and nuanced view of how families from heterogeneous social environments experience ASD.

Thirdly, the sample size was limited and children with formally diagnosed comorbidities were excluded. Finally, most respondents were mothers or female caregivers, so the results might not generalize to fathers or male caregivers. Future studies should incorporate more fathers or male caregivers to compare perspectives and assess differing impacts on FQoL.

### Clinical Implications

The results of the present study highlight several clinical implications for interventions seeking to enhance FQoL in children with ASD and their families. For example, addressing sleep and mealtime difficulties in conjunction with core ADS features may significantly bolster FQoL. While all forms of sleep and eating challenges appeared to affect FQoL, certain factors such as sleep anxiety, daytime sleepiness, and food refusal may merit particular attention, without disregarding other pertinent difficulties. Specifically, interventions should consider both pharmacological (such as melatonin for sleep disorders; Nogueira et al., 2023 for a meta-analysis) and nonpharmacological approaches, tailored to the individual needs and preferences of the child and family. Local governments should also promote community-based programs that support parents in fostering healthier lifestyle for their children with ASD. Providing strategies to manage and mitigate difficulties (e.g., establishing consistent bedtime routines or creating a sleep-friendly environment for sleep challenges; and creating structured environments, or offering positive reinforcement and advice on feeding therapy for mealtime challenges) could alleviate caregiver burden, thereby improving FQoL.

Building on these findings, our results provide a guideline for healthcare professionals, educators, and families to work together to design high-impact interventions that target sleep and mealtime challenges in children with ASD, leading to tangible improvements in FQoL. Specifically, these insights can inform the creation of practical workshops, accessible guides, and tailored psychoeducational resources for parents and educators. These resources should equip parents and educators with evidence-based strategies, to establish adapted sleep and feeding routines that are sensitive to the unique needs of children with ASD. Identifying and addressing these modifiable behaviors can disrupt the cycle between disrupted activities of daily living, caregiver stress, and decreased family well-being. This can not only improve developmental prognosis and adaptive functioning in children with ASD, but also lead to more harmonious family functioning, lower level of parental burnout, and a better sense of well-being for all family members.

## Conclusions

This study investigated the impact of several health-related behaviors on FQoL in children with ASD, demonstrating that sleep and mealtime problems can pose significant risks to FQoL independently of screen time, physical activity, gender, age, and autistic traits. In conclusion, the current work highlights the importance of targeting specific sleep and feeding difficulties to improve the FQoL of children with ASD. Although more research on aspects like screen time and physical exercise is needed, the results offer a sound basis for developing clinical and community interventions which will help both kids and their families.
